# 4-Coumarate-CoA Ligase-Like Gene *OsAAE3* Negatively Mediates the Rice Blast Resistance, Floret Development and Lignin Biosynthesis

**DOI:** 10.3389/fpls.2016.02041

**Published:** 2017-01-10

**Authors:** Hao Liu, Zhenhua Guo, Fengwei Gu, Shanwen Ke, Dayuan Sun, Shuangyu Dong, Wei Liu, Ming Huang, Wuming Xiao, Guili Yang, Yongzhu Liu, Tao Guo, Hui Wang, Jiafeng Wang, Zhiqiang Chen

**Affiliations:** ^1^National Engineering Research Center of Plant Space Breeding, South China Agricultural UniversityGuangzhou, China; ^2^Department of Rice Breeding, Jiamusi Rice Research Institute of Heilongjiang Academy of Agricultural SciencesJiamusi, China; ^3^Department of Plant Breeding, College of Agricultural, South China Agricultural UniversityGuangzhou, China; ^4^Plant Protection Research Institute Guangdong Academy of Agricultural Sciences/Guangdong Provincial key Laboratory of High Technology for Plant ProtectionGuangzhou, China

**Keywords:** 4CL-like, Rice blast, AMPBP, Lignin, ROS

## Abstract

Although adenosine monophosphate (AMP) binding domain is widely distributed in multiple plant species, detailed molecular functions of AMP binding proteins (AMPBPs) in plant development and plant-pathogen interaction remain unclear. In the present study, we identified an AMPBP *OsAAE3* from a previous analysis of early responsive genes in rice during *Magnaporthe oryzae* infection. *OsAAE3* is a homolog of Arabidopsis *AAE3* in rice, which encodes a 4-coumarate-Co-A ligase (4CL) like protein. A phylogenetic analysis showed that *OsAAE3* was most likely *4CL-like 10* in an independent group. *OsAAE3* was localized to cytoplasm, and it could be expressed in various tissues. Histochemical staining of transgenic plants carrying *OsAAE3* promoter-driven GUS (β-glucuronidase) reporter gene suggested that *OsAAE3* was expressed in all tissues of rice. Furthermore, *OsAAE3-OX* plants showed increased susceptibility to *M. Oryzae*, and this finding was attributable to decreased expression of pathogen-related 1a (*PR1*) and low level of peroxidase (POD) activity. Moreover, *OsAAE3* over-expression resulted in increased content of H_2_O_2_, leading to programmed cell-death induced by reactive oxygen species (ROS). In addition, *OsAAE3* over-expression repressed the floret development, exhibiting dramatically twisted glume and decreased fertility rate of anther. Meanwhile, the expressions of lignin biosynthesis genes were significantly decreased in *OsAAE3-OX* plants, thereby leading to reduced lignin content. Taken together, *OsAAE3* functioned as a negative regulator in rice blast resistance, floret development, and lignin biosynthesis. Our findings further expanded the knowledge in functions of AMBPs in plant floret development and the regulation of rice-fungus interaction.

## Introduction

Adenosine monophosphate (AMP) binding domain-containing proteins widely exist in various plant species, and this family consists of members with diverse functions, including luciferases, peptide antibiotic synthetases, acetyl-CoA synthetases (ACSs), acyl-CoA synthetases, 4-coumarate-CoA ligases (4CLs), and various other closely-related synthetases (Shockey et al., 2000; Stremmel et al., 2001; Shockey and Browse, 2011). Members of this superfamily catalyze the initial adenylation of a carboxylate to form an acyl-AMP intermediate, followed by a secondary partial reaction, most commonly the formation of a thioester (Can et al., 2014). ACSs play a crucial role in both de novo synthesis and modification of existing lipids, and the resulting products also participate in the regulation of plant growth and development (Sasaki and Nagano, 2004; Souza et al., 2008). AMP-binding domain-containing 4CLs are critical enzymes in the phenylpropanoid metabolism pathway, which drive the carbon flow from primary metabolism to different branches of secondary metabolism in plant. 4CLs in Arabidopsis have overlapping and distinct roles in phenylpropanoid metabolism. *4CL1* accounts for the majority of the total 4CL activity, and loss of *4CL1* leads to reduced lignin content without growth defect (Soltani et al., 2006). *4CL3* is expressed in a broad range of cell types, and it probably has an extra function in flavonoid metabolism. In addition, free fatty acids released from the plastids become metabolically available when they are converted to their corresponding Co-A thioesters (Li et al., 2015). This activation is induced by long-chain acyl-coenzyme asynthetases (LACSs). *LACS4* and *LACS9* double-mutants have shown to strongly reduce biosynthesis of endoplasmic reticulum-derived lipid precursors, which are necessary substrates for glycolipid synthesis in the plastids (Jessen et al., 2011). The expression of rice *OsBIABP1* is activated by *M. oryzae* infection, which may be a defense-related AMP-binding protein (AMPBP) that is involved in the regulation of defense response through SA and/or JA/ET signaling pathways. However, functions of those genes remain unexplored in rice (Zhang et al., 2009).

As a polyphenolic polymer, lignin is accumulated and deposited in cell wall, and this accumulation enhances the ability of the cell wall and provides mechanized protection for the plasma membrane-wrapped protoplasm (Zhao, 2016). However, the lignin deposition is highly dependent on the cell type, tissue, developmental stage and plant species. As part of the normal differentiation and function of specific cell types, lignification also serves as an integral feature of restriction to plant non-woody tissues (Barros et al., 2015). Lignin biosynthesis can be triggered as a response to various biotic and abiotic stresses in cells. Evidence has clearly illustrated that lignin biosynthesis genes play crucial roles in basal defense and normal growth of plants (Wang and Balint-Kurti, 2016). PALs can catalyze the lignin precursor phenylalanine and transform it into cinnamic acid in lignin biosynthesis pathway (Pascual et al., 2016). *OsPAL4* is able to improve broad-spectrum disease resistance in rice by increasing the expression of *OsPAL2* and repressing the expression of unlinked *OsPAL6* (Tonnessen et al., 2015). Cinnamyl alcohol dehydrogenase (CAD) catalyzes the last step of monolignol biosynthesis. *OsCAD2* is largely responsible for monolignol biosynthesis in rice stem, while mutant plant exhibits drastically reduced CAD activity and undetectable sinapyl alcohol dehydrogenase activity (Zhang et al., 2006; Hirano et al., 2012). 4CL mediates the activation of a number of hydroxycinnamic acids for the biosynthesis of monolignols and other phenolic secondary metabolites in higher plants. Suppression of *Os4CL3* expression results in significant lignin reduction, impaired plant growth, decreased panicle fertility and other morphological changes (Gui et al., 2011).

Rice blast is caused by the ascomycetous fungus *Magnaporthe oryzae*, which is one of the most serious and devastating epiphytic diseases in rice production worldwide (Ashkani et al., 2015). Currently, more than 24 major *R* genes conferring resistance to *M. oryzae* have been identified in rice, including *Pi-ta* (Jia et al., 2016), *Pi-k* (Wu et al., 2014), and *Pb1* (Inoue et al., 2013), and modulation of these *R* genes significantly maintains and improves the grain yield and quality in modern rice cultivars. These major R genes mainly encode the nucleotide-binding site-leucine-rich repeat (NBS-LRR) proteins that recognize diverse effectors (Avirulence proteins, Avr) and activate the downstream immunity response (DeYoung and Innes, 2006; Marone et al., 2013). Meanwhile, the rice-blast system has been developed as a model to study the mechanism of pathogen-associated molecular pattern-triggered immunity (PTI) and effector-triggered immunity (ETI) in plant-fungus interaction (Andolfo and Ercolano, 2015; Stael et al., 2015). To date, the underlying molecular mechanism of rice resistance to diseases has been illustrated in multiple levels, including transcriptome, proteome, post-transcriptional modification and epigenetic regulation (Miah et al., 2013; Xu et al., 2015; Li et al., 2016; Sharma et al., 2016). Various regulatory factors mediating the blast resistance in rice have been identified by a combination of biochemical, genetic and high-throughput sequencing approaches. However, functional roles of single gene in complex defense network of rice blast still need to be further elucidated.

In the present study, we identified and characterized an Arabidopsis *AAE3* (Foster et al., 2012) homolog in rice. Arabidopsis *AAE3* encodes a specific cytoplasmic oxalyl-CoA synthetase containing the conserved AMP-binding domain, which is required for oxalate degradation, normal seed development and defense against an oxalate-producing fungal pathogen. Here, the cytoplasmic 4CL like protein OsAAE3 (LOC_Os04g58710) was identified from an analysis of transcriptome and proteome profile. In leaf tissue, increased *OsAAE3* activity was significantly correlated with decreased resistance to rice blast and reduced lignin content. Furthermore, our results also showed that *OsAAE3* possessed multiple potential roles in metabolism and plant anther development.

## Materials and methods

### Plant materials and growth conditions

*Oryzae sativa* japonica cultivar *Pik-H4* NILs was used as the wild-type rice strain in this study. *Pik-H4* NIL contains the *Pik-H4* resistance gene (an allele of *Pik* locus) in the susceptible cultivar LTH background. The *M. oryzae* race *GDYJ7*, one of the primary *M. oryzae* races found in Guangdong Province, China, is incompatible with *Pik-H4*.

Sixth-leaf-stage rice seedlings were used in the present study, which were grown under natural light in a greenhouse at 26°C for inoculation of the rice blast fungus. Freshly prepared *M. oryzae* spores (1 × 10^5^ conidia/mL 0.02% v/v gelatin) were sprayed onto the rice leaves using an air sprayer. Inoculated plants were kept in a humidity chamber at 28°C, and rice leaves were harvested for RNA extraction at 0, 12, 24, 36, and 48 h after inoculation.

### Subcellular localization analysis

The full-length *OsAAE3* cDNA insert without a stop codon was amplified by PCR. Amplified fragments were digested with *Xba*I/*Bam*HI and cloned between the cauliflower mosaic virus (CaMV) 35S promoter and the GFP gene. Rice protoplast was isolated from 12-day-old rice seedling stem and sheath. Briefly, 30 rice seedlings were cut into approximately 0.5 mm strips, and then incubated in an enzyme solution (1.5% Cellulase RS, 0.75% Macerozyme R-10, 0.6 M mannitol, 10 mM MES at pH 5.7, 10 mM CaCl_2,_ and 0.1% BSA) for 4–5 h in the dark with gentle shaking (60–80 rpm). After washing twice with W5 solution (154 mM NaCl, 125 mM CaCl_2_, 5 mM KCl, and 2 mM MES at pH 5.7), last protoplast was resuspended in MMG solution (0.4 M mannitol, 15 mM MgCl_2,_ and 4 mM MES at pH 5.7). The resulting OsAAE3-GFP fusion construct and empty GFP vector were transiently co-expressed in rice protoplasts by 40% PEG induction. Fluorescence was examined using laser-scanning confocal microscope (Zhang et al., 2011) (Model LSM 780; Carl Zeiss, Jena, Germany).

### GUS assay

We first cloned about 2 kb promoter region of OsAAE3 from rice genomic DNA. The amplified sequence was inserted into the *Nco*I/*Bam*HI sites of pCAMBIA1305 vector. The resulting construct was introduced into agrobacterium strain EHA105 and transformed to wild-type (*Pik-H4* NIL) calli as described previously. The tissues of the transgenic plants were washed three times with 100 mM NaPO_4_ buffer (pH 7.0), and incubated with a staining solution [100 mM NaPO_4_ (pH 7.0), 10 mM EDTA, 2 mM 5-bromo-4-chloro-3-indolyl-b-GlcA, 5 mM K_4_Fe(CN)_6_, 5 mM K_3_Fe(CN)_6_, and 0.2% Triton X-100] for 20 min to 24 h at 37°C (Jefferson et al., 1987).

### Total RNA extraction and real-time PCR analysis

Total RNA was extracted from 100 mg of fourth-leaf-stage rice seedling with Trizol Reagent (Invitrogen, Beijing, China), and purified RNA was reversely transcribed into cDNA using PrimeScript RT reagent Kit (Takara, Dalian, China) according to the manufacturer's instructions. The cDNA was quantified by real-time PCR using a 20 μL reaction system by SYBR Premix ExTaq™ (TaKaRa, Dalian, China) on an ABI StepOne Plus system. Table S3 lists the primer sequences used for PCR analysis. Differences in gene expression were expressed as fold change relative to control and calculated using the 2^−Δ*ΔCT*^ method. Each measurement was carried out in triplicate, and the error bars represent SE of the mean of fold changes for three biological replicates.

### Generation of the *OsAAE3-OX* transgenic plants

The full-length of *OsAAE3* cDNA was isolated by RT-PCR from the leaves of fourth-leaf-stage rice plants using the cDNA F/R primers (Table S3) encompassing the translation start and stop codons. This cDNA insert was digested with *Bam*HI and cloned between the maize ubiquitin promoter and the *Nos* terminator in the plant expression vector pOX containing the hygromycin resistance gene as a selection maker. Prof. Yaoguang Liu (South China Agricultural University, Guangzhou 510642, China) provided the plant binary vector pOX. pOX*-OsAAE3* was then introduced into agrobacterium strain EHA105 and then transformed to wild-type (*Pik-H4* NIL) calli as described previously (Hiei et al., 1994). Transgenic rice plants were regenerated from the transformed calli on selection medium containing 50 mg/L hygromycin and 250 mg/L cefotaxime. *OsAAE3* levels in the transgenic rice plants were further confirmed with real-time PCR.

### Measure POD activities and H_2_O_2_ content in fresh leaves

The enzyme extracts of POD were prepared following the method of Cai et al. (2008) with some modifications. Briefly, 300 mg fresh leaves were frozen and ground in liquid N_2_, and the powder was mixed with 4 mL 0.05 M PBS (pH 7.8) and transferred into 5-ml tube. After thawing, the tubes were centrifuged at 8000 rpm/min for 15 min, and the supernatant containing the total peroxidase (POD) was collected. The POD activity was measured as the rate of decomposition of H_2_O_2_ by POD, with guaiacol as the hydrogen donor, by spectrophotometrically measuring the rate of color development at 436 nm due to guaiacol oxidation (Cai et al., 2008).

Hydrogen peroxide was performed using the Ferric Xylenol Orange method as described previously. Actually, fresh leaf tissue was ground in cold acetone and filtered to remove cellular debris. The supernatants were extracted with CCl4-CHCl3 solution. Then the extract was transferred into a new tube containing 250 μM ferrous ammonium sulfate, 100 μM sorbitol and 100 μM xylenol orange in 25 mM H_2_SO4. The mixture reacted 30 min in the dark at room temperature, and the absorbance was detected at 560 nm (Gay et al., 1999).

### Lignin content assay

Briefly, 1 g of fresh leaves was homogenized in 5 mL cold 95% ethanol and centrifuged at 5000 rpm/min for 30 min, and the precipitate was washed by ethanol-hexane solution (1:2, V/V) for three times. After thoroughly dried, the washed precipitate was placed in a glass reaction vial (15 mL) with 5 mL of 25% (v/v) acetyl bromide in acetic acid, sealed with Teflon lined caps, and heated at 70°C for 30 min. After digestion, the vial's contents were quantitatively transferred to a 10-mL volumetric flask containing 0.9 mL of 2 M NaOH, 5 mL of acetic acid and 0.1 mL of 7.5 M hydroxylamine, and the flask was filled to 10 mL with acetic acid. After reaction solution was centrifuged at 1000 g for 7 min, the absorption values of supernatant were determined at 280 nm. According to the standard curve, lignin contents were calculated (Xie et al., 2011).

## Results

### OsAAE3 expression induced by *M. oryzae*

**Figure 1:**
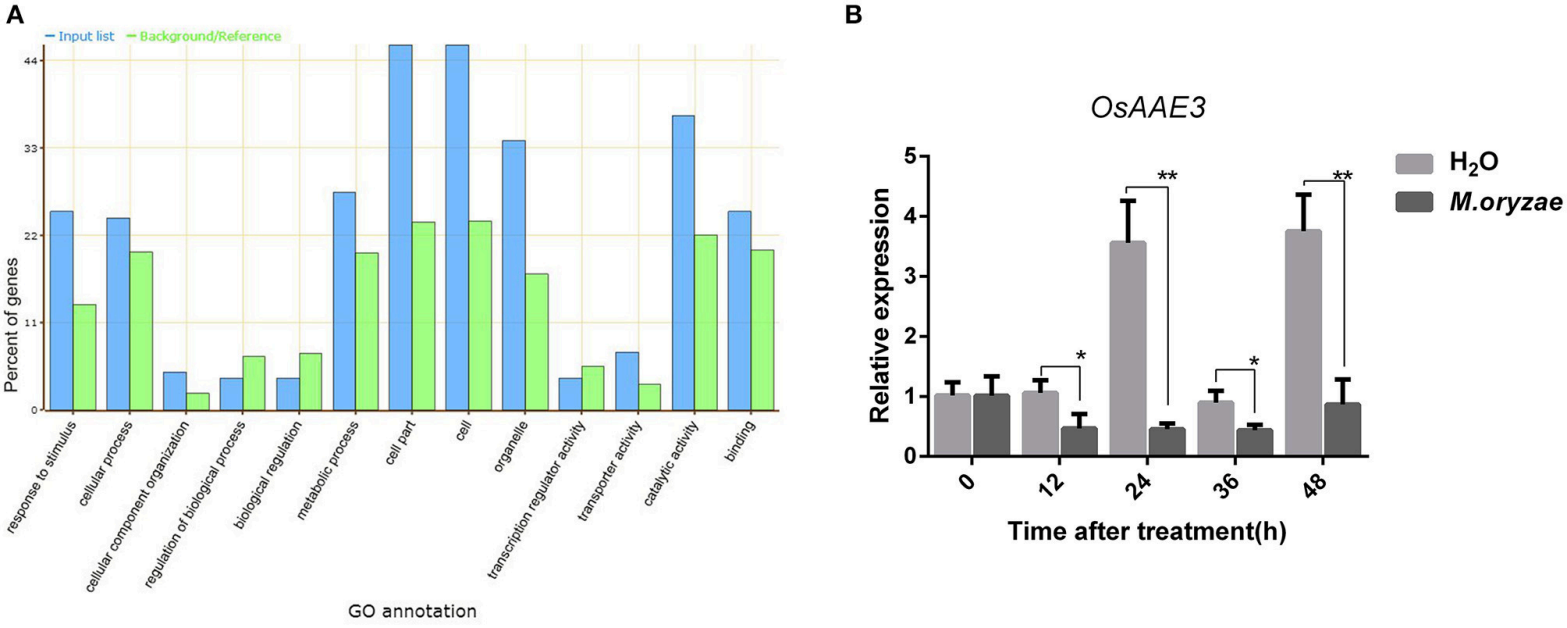
*****OsAAE3*** expression was down-regulated by ***M. oryzae***. (A)** GO annotation of differential genes expression in *Pik-H4* NILs by transcription-proteomics analysis. **(B)** OsAAE3 expression induced by *M. oryzae* after 48 h, Sterile H_2_O was used as controls. Values shown are means ± SD from three independent experiments, and asterisks indicate a significant difference according to the *t*-test (*P* < 0.05) compared with control group.

We have previously compared the global gene expression in resistance line *Pik-H4* NILs with the susceptible cultivar LTH after *M. oryzae* inoculation *via* a transcriptome-proteome analysis. We identified 61 and 69 genes that were up-regulated and down-regulated in *Pik-H4* NILs line, respectively (Table S1). Based on our transcriptome-proteome analysis in *Pik-H4* NILs line, *Pik-H4* modulates multiple genes involved in diverse biological processes, including defense-related hormone biosynthesis, disease resistance, response stress, photosynthesis, and signal transduction (Figure 1A).

An AMPBP3 (4CL-like protein), named OsAAE3 (LOC_Os04g58710), was identified from the transcriptome and proteome analysis of early responsive genes in rice during *M. oryzae* infection (Table S1). We then examined the expression pattern of *OsAAE3* over a time course of 48 h after inoculation with *M. oryzae* by quantitative RT-PCR (qRT-PCR). The *OsAAE3* expression at the mRNA level was significantly decreased at 12 h and reached its lowest level at 24 h, and then it was maintained at a relatively low level from 24 to 48 h after inoculation with *M. oryzae* in wild-type plants (Figure 1B). In contrast, the *OsAAE3* expression presented circadian rhythmicity pattern during the whole stage after spraying of water.

### Identification and characterization of *OsAAE3*

**Figure 2:**
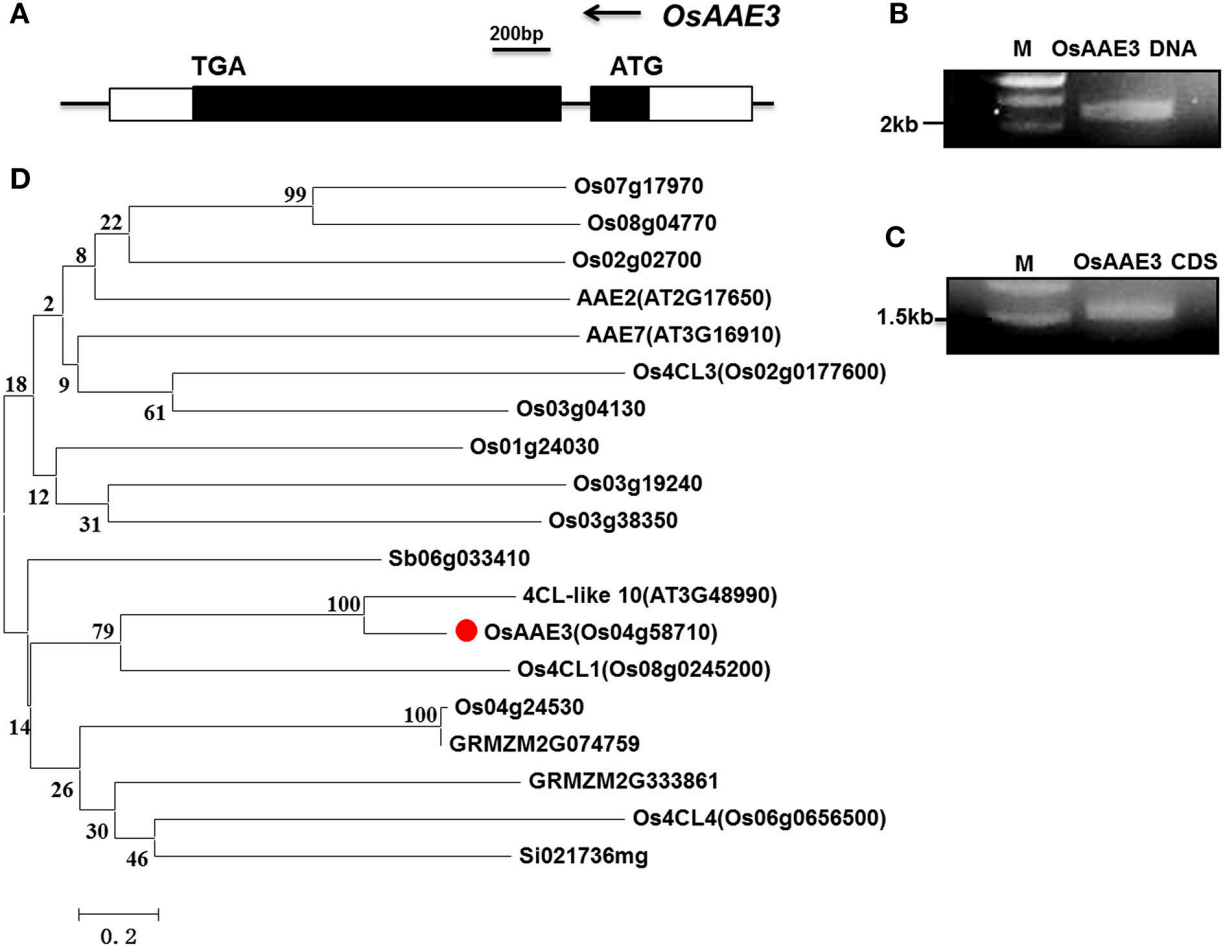
**Identification and characterization of ***OsAAE3*****. **(A)** Schematic gene structure of *OsAAE3* in rice genome. **(B,C)** The OsAAE3 DNA and CDS fragments were examined by agarose gel electrophoresis. **(D)** Comparative phylogenetic analysis of the OsAAE3 proteins in plants. Sequences were aligned using ClustalX. The evolutionary history was inferred using a Neighbor-Joining phylogenetic tree generated with the software MEGA6. The percentage of replicate trees in which the associated taxa clustered together in the bootstrap test (2000 replicates) is shown next to each branch. Putative OsAAE3 (Os04g58710) members in *A. thaliana* (AT2G17650, AT3G16910, AT3G48990), *O. sativa* (Os07g17970, Os08g04770, Os02g02700, Os02g0177600, Os03g04130, Os01g24030, Os03g19240, Os03g38350, Os08g0245200, Os04g24530, Os06g0656500), *Sorghum bicolor* (Sb06g033410), *Zea mays* (GRMZM2G074759, GRMZM2G333861), *Setaria italica* (Si021736 mg).

The genome sequence and the cDNA fragment encoding of *OsAAE3* were isolated from rice using gene-specific primers based on the sequence (LOC_Os04g58710) of the rice genome database (Rice Genome Annotation Project). The genome sequence of OsAAE3 was 2221 bp, full-length coding sequence (CDS) was 1557 bp, which harbored two exons and one intron, and it encoded a protein of 519 amino acid residues with a deduced molecular weight of 54.47 kDa. This result was similar to our DNA sequence analysis (Figures 2A–C).

According to the prediction from Pfam database and comparison with other AMPBPs, amino acid sequence analysis showed that an AMP-binding domain was located from 34 to 439 aa in the OsAAE3 sequence. Furthermore, we identified orthologous protein sequence of *OsAAE3* from several plant models, including rice, Arabidopsis, maize, sorghum and soybean. A phylogenetic analysis based on those sequences showed that *OsAAE3* was most likely *4CL-like 10* in an independent group (Figure 2D). As indicated from the sequence alignment, there were 90% similarities between *OsAAE3* and *4CL-like 10* (Figure S1). Therefore, it is of great interest to verify whether *OsAAE3* was indeed involved in monolignol catabolism in the same way as its homolog in Arabidopsis.

### OsAAE3 is localized to the cytosol

**Figure 3:**
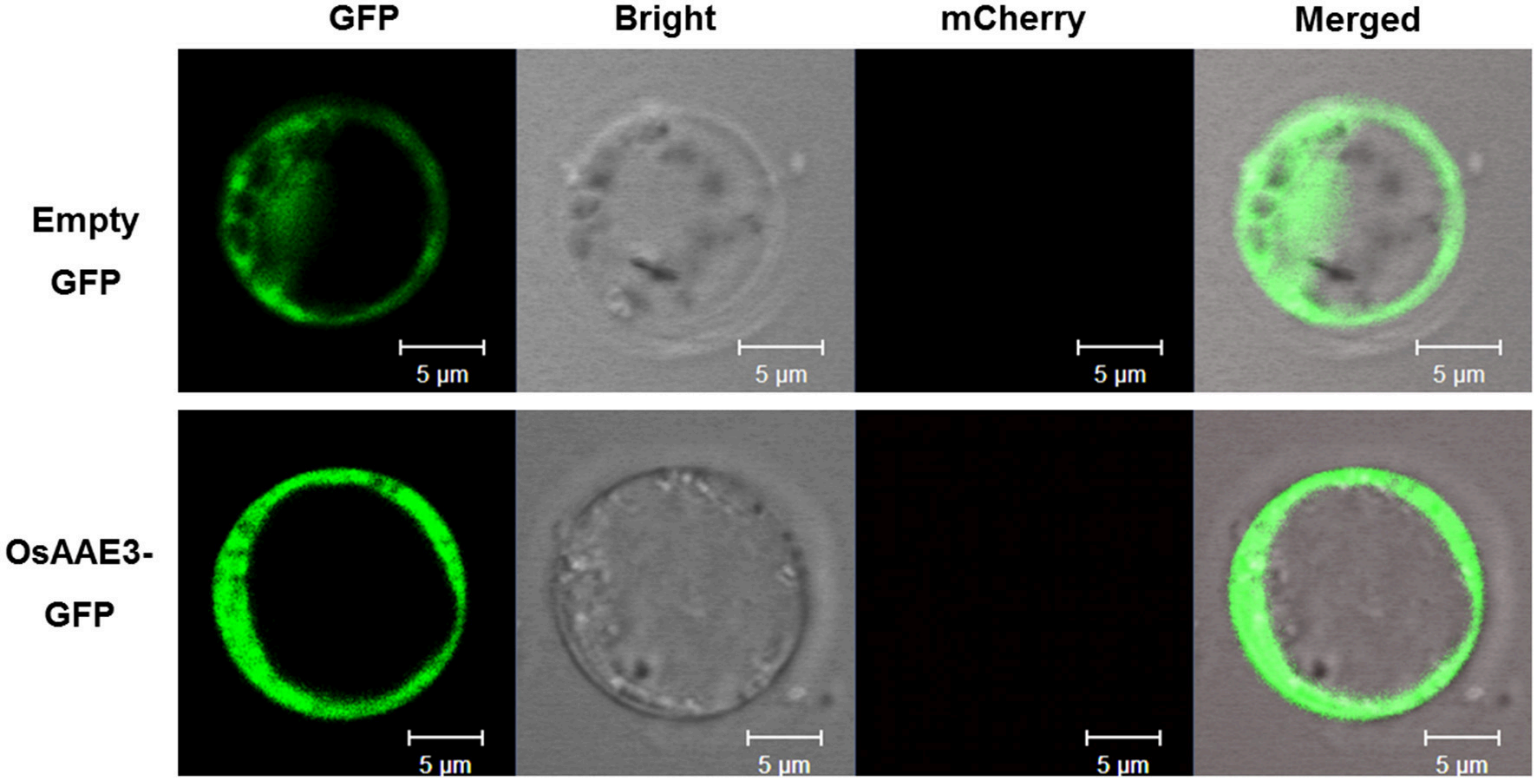
**Subcellular localization assay of OsAAE3**. Subcellular localization indicated that OsAAE3-GFP signal was strongly detected in the cytoplasm of rice protoplast cell. OsAAE3-GFP and GFP signals are green, Scale bar is 5 μm.

The AMP-binding domain of OsAAE3 suggested that it was probably localized to the cytosol like its homolog AAE3 in Arabidopsis. Meanwhile, the PSORT database revealed the multi-organelle localization of OsAAE3, including the mitochondrial inner membrane, plasma membrane, Golgi body, and mitochondrial intermembrane space, but the available prediction scores were unreliable. To further investigate the subcellular localization of OsAAE3, we constructed an OsAAE3-GFP fusion protein driven by the CaMV 35S promoter, and the empty GFP was used as the negative control. The resulting vectors 35S:OsAAE3-GFP and GFP were transiently co-transformed into rice protoplast cells with the PEG-mediated procedure. Interestingly, the OsAAE3-GFP fusion protein exhibited similar pattern to the empty GFP control, and the OsAAE3-GFP signal was strongly detected in the cytoplasm of rice protoplast cells (Figure 3). Therefore, the transient expression assay indicated that *OsAAE3* encoded a cytoplasmic synthetase like 4CL that could possibly catalyze the lignin degradation.

### Expression pattern analysis of *OsAAE3*

**Figure 4:**
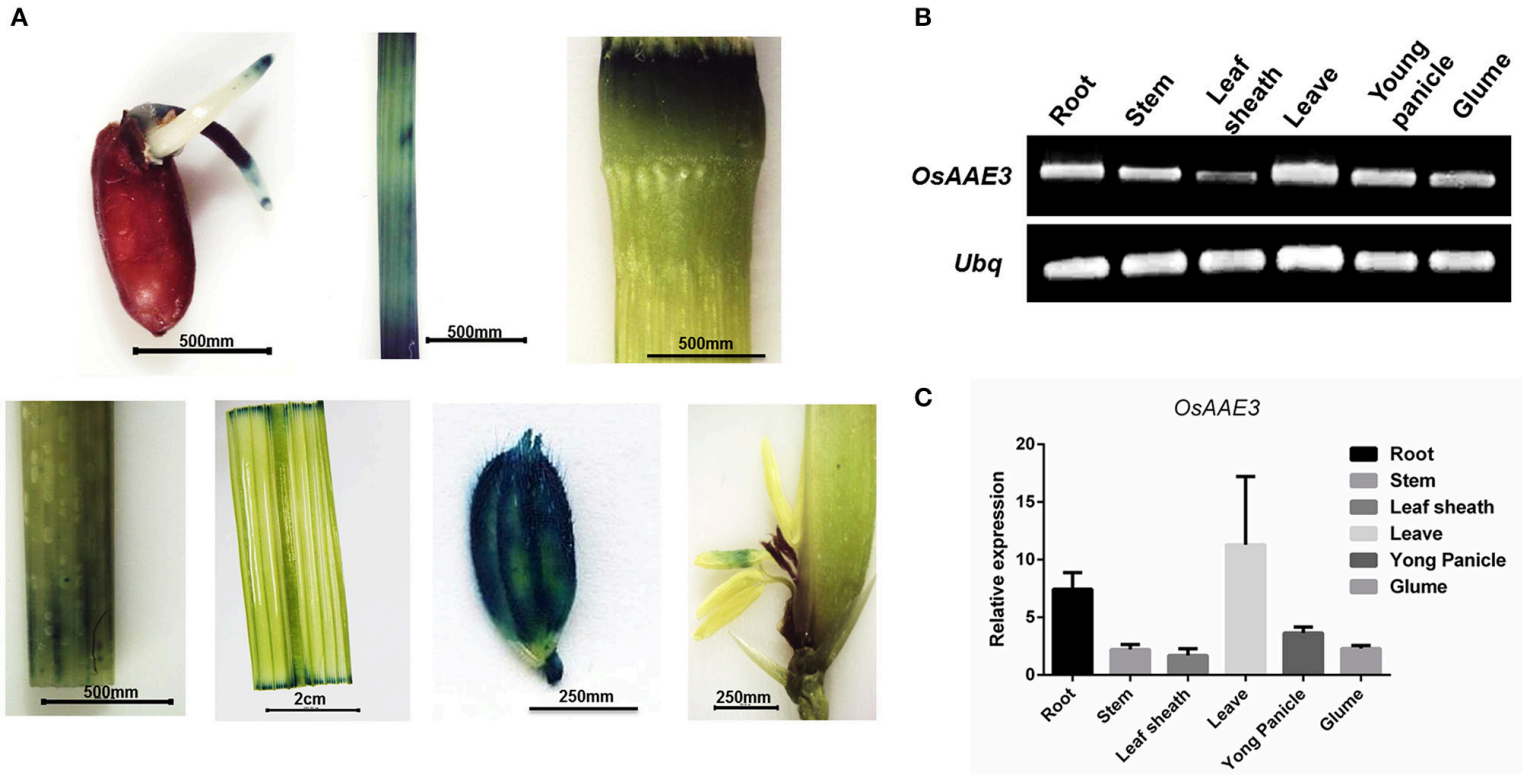
**Expression pattern analysis of ***OsAAE3***. (A)** Activity determination of the *OsAAE3* promoter. GUS staining of germinated seed, stem, node, shoot, blade leaf, glume, and anther. **(B,C)** Semi-quantitative PCR and real-time PCR determined the relative expression of *OsAAE3* in root, stem, leaf sheath, leave, young panicle, and glume.

To evaluate the expression pattern of *OsAAE3* in different tissues, total RNA was extracted from root, stem, leaf sheath, leaf blade, young panicle and glume of rice at the heading stage. Semi-quantitative PCR and real-time PCR (RT-PCR) were performed to determine the relative expression of *OsAAE3*. Semi-quantitative PCR results suggested that the full-length coding sequence of *OsAAE3* could be easily amplified from all of tissues without tissue specificity (Figure 4B). *OsAAE3* was constitutively expressed in various types of tissues, but its highest expression was detected in leaf blade, followed by root, young panicle, glume, stem, and leaf sheath. Such finding was confirmed by RT-PCR experiment (Figure 4C).

We further cloned the promoter sequence of *OsAAE3* into GUS (β-glucuronidase) reporter system, and then the constructed promoter (*OsAAE3*)-GUS fusion vector was transformed into rice callus to assess the promoter activity. GUS staining analysis indicated that the *OsAAE3* promoter activity was detected in the root, epidermis cells of leaves, stem vascular cells, glume vascular cells and anther (Figure 4A). Taken together, *OsAAE3* was expressed in all tissues of the plant.

### Generation of the *OsAAE3-OX* transgenic plants

**Figure 5:**
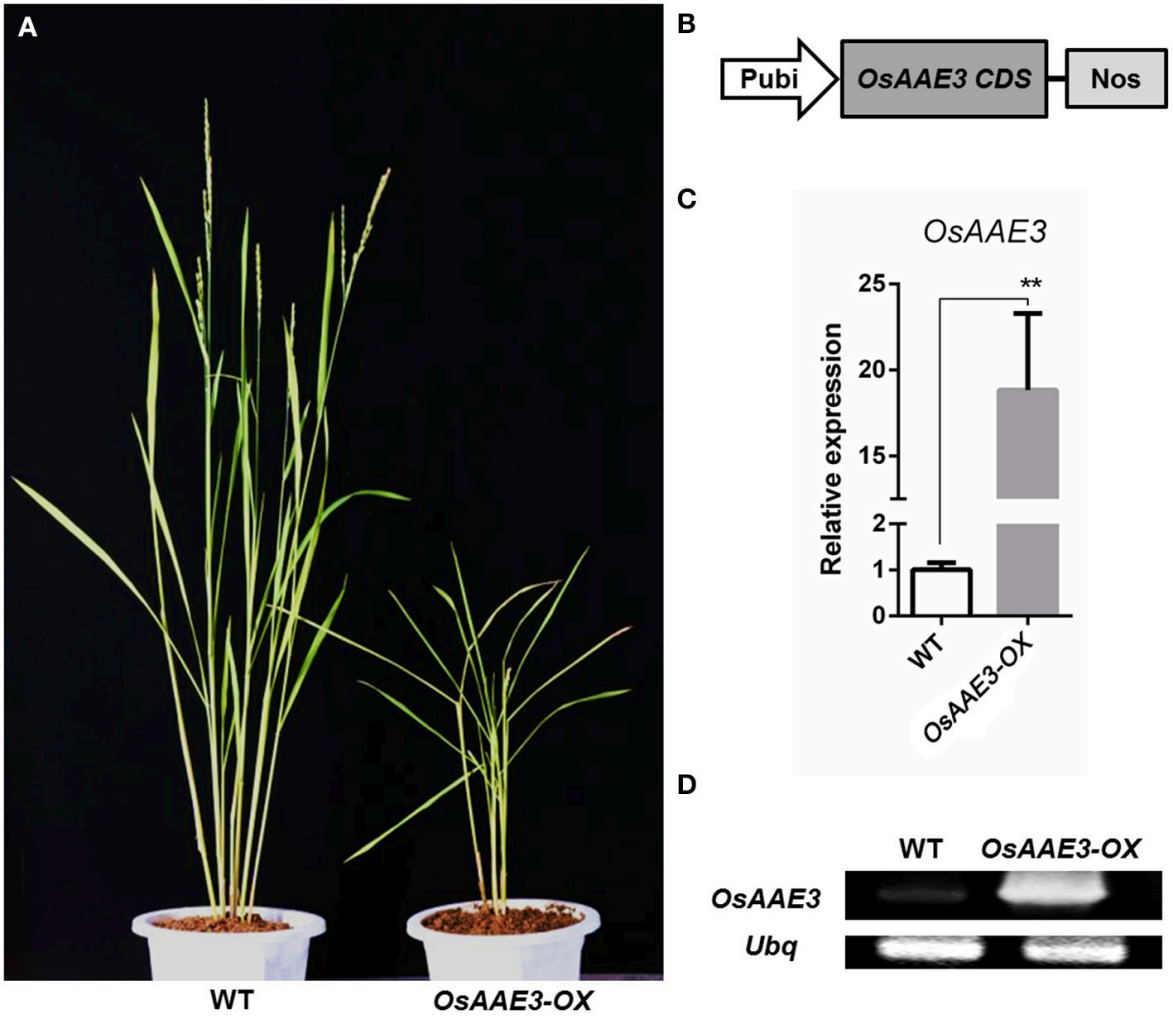
*****OsAAE3*** transgenic plants Identification. (A)** The morphology of wild-type and *OsAAE3-OX* transgenic plants (T1 generation). The photograph was taken about 60 days after heading of *OsAAE3* transgenic plants (single line) and wild-type plants. **(B)** Schematic structure of OsAAE3-OX transgenic vectors construction. **(C,D)** Expression analysis of *OsAAE3* overexpression line measured by semi-quantitative PCR and real-time PCR compared with wild-type. Values shown are means ± SD from three independent experiments, and asterisks indicate a significant difference according to the *t*-test (*P* < 0.05) compared with WT.

To determine the detailed molecular roles of *OsAAE3* in rice physiological and biochemical reactions, we constructed the transgenic rice plants over-expressing *OsAAE3* (*OsAAE3-OX*) under the control of ubiquitin promoter (Figure 5B). The *OsAAE3* expression at the mRNA level was significantly increased in transgenic plants (*OsAAE3-OX*) compared with wild-type plants, and these data were validated using qRT-PCR and semi-quantitative PCR (Figures 5C,D). Over-expression of *OsAAE3* repressed the plant growth, showing dwarfing, rolling, and narrow leaves as well as abnormal glumes (Figure 5A and Figure S2). Therefore, based on the observed abnormal phenotypes of *OsAAE3-OX* plants, we believed that *OsAAE3* served as a negative regulator in the regulation of plant development and growth.

### Over-expression of *OsAAE3* reduces the rice blast resistance

**Figure 6:**
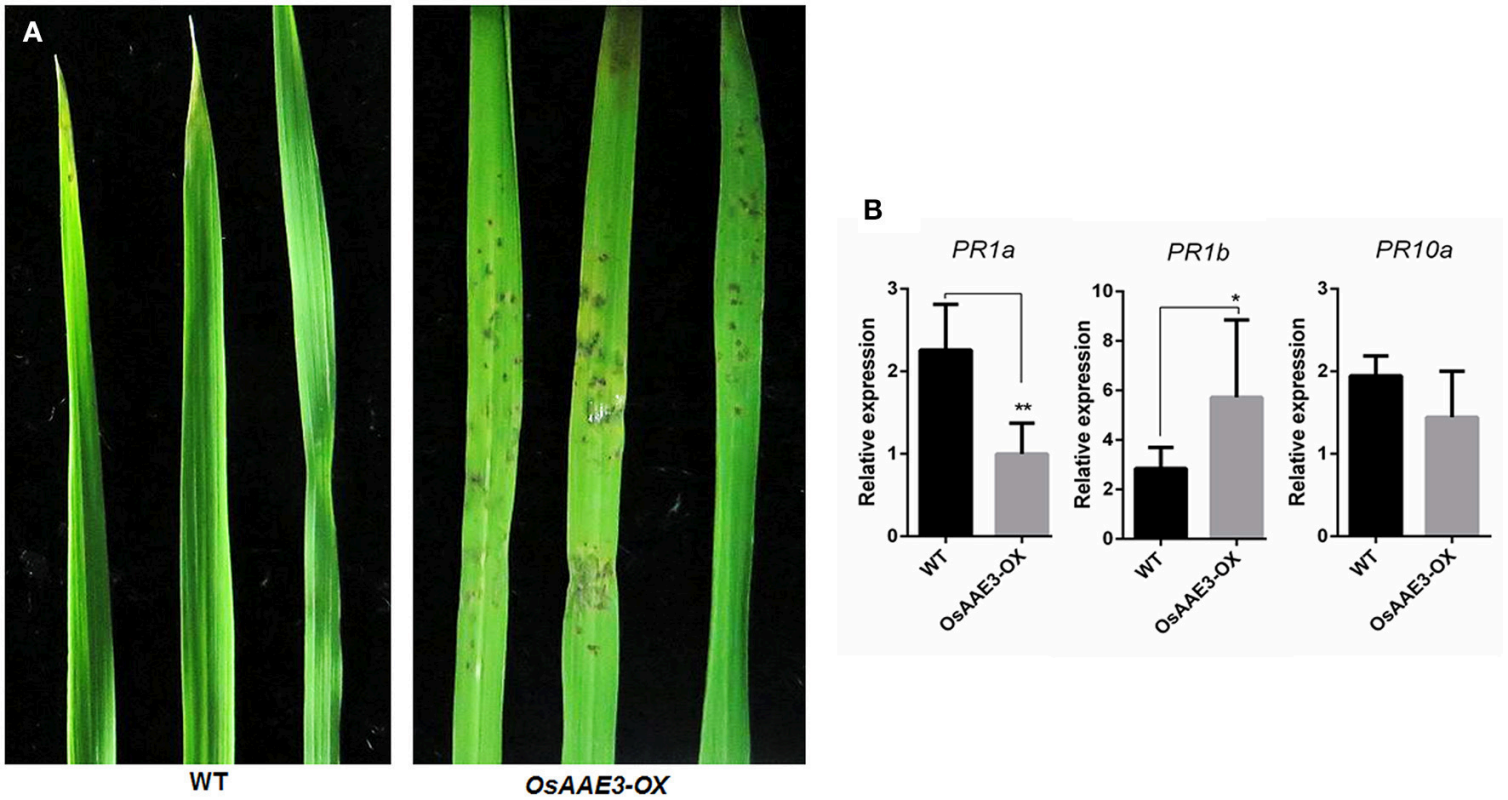
*****OsAAE3*** reduced the rice blast resistance. (A)** Photographs of blast fungus-inoculated fourth leaves of *OsAAE3* transgenic plants and wild-type at the six-leaf stage. A conidial suspension of blast fungus race GDYJ7 was sprayed on the leaf surfaces, and local lesions were observed 5 days later. **(B)** Relative expression of *PR1a, PR1b*, and *PR10* in *OsAAE3-OX* and wild-type plants. Values shown are means ± SD from three independent experiments, and asterisks indicate a significant difference according to the *t*-test (*P* < 0.05) compared with WT.

We next examined resistance of the transgenic plants to *M. oryzae* in order to elucidate the molecular basis of *OsAAE3* in disease resistance in rice. Disease symptoms in plants were quantified at 7 days after inoculation, and the *OsAAE3-OX* plants exhibited reduced resistance to *M. oryzae* compared with wild-type plants (Figure 6A). This result indicated that over-expression of *OsAAE3* affected the basal level of resistance to *M. oryzae* in rice. In addition, the disease spots on the leaf surface were intensively associated with expressions of pathogen-related (*PR*) genes. We further investigated expressions of three *PR* genes in *OsAAE3-OX* and wild-type plants by RT-PCR. The data demonstrated that the expressions of *PR1a* (Mitsuhara et al., 2008) and *PR10* (Choi et al., 2015) were down-regulated in *OsAAE3-OX* plants compared with wild-type pants under the normal growth condition, but the *PR10* expression was not significantly decreased in *OsAAE3-OX* line (Figure 6B). However, the expression of *PR1b* (Agrawal et al., 2000) was significantly increased in *OsAAE3-OX* plants (Figure 6C). Therefore, we concluded that blast induced by *OsAAE3* over-expression was attributable to the reduced PR1a protein that down-regulated the innate defense response upon fungal invasion.

**Figure 7:**
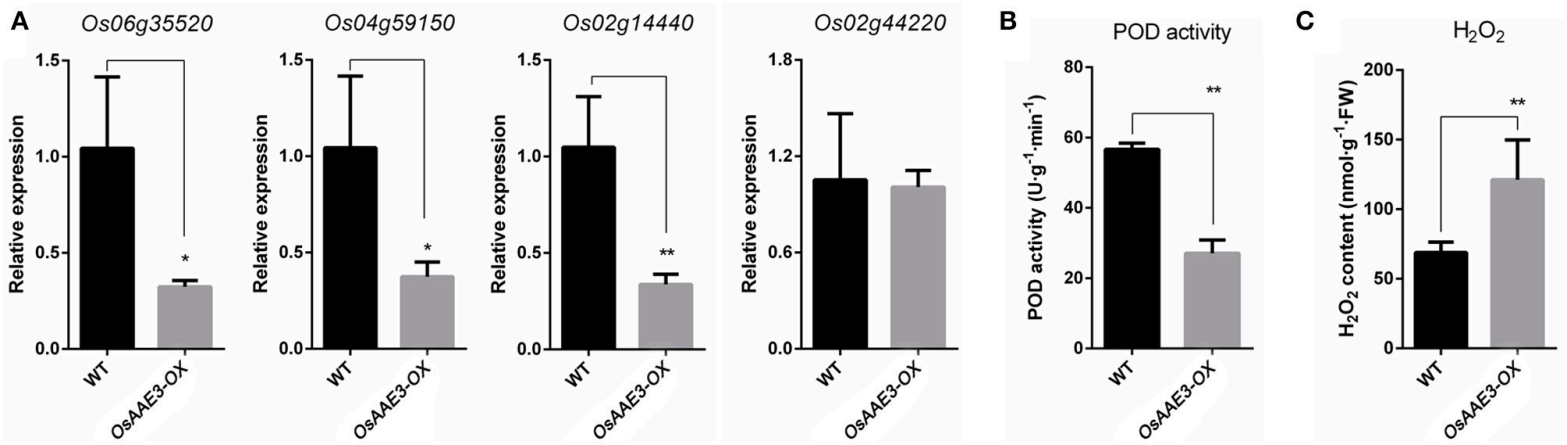
*****OsAAE3*** negatively regulates POD activity during the ROS accumulation. (A)** Relative expression of POD synthesis-related genes in *OsAAE3-OX* and wild-type plants. Values shown are means ± SD from three independent experiments, and asterisks indicate a significant difference according to the *t*-test (*P* < 0.05) compared with WT. **(B,C)** POD activity assay and H_2_O_2_ content assay in *OsAAE3-OX* and wild-type plants. Values shown are means ± SD (*n* = 10), and asterisks indicate a significant difference according to the *t*-test (*P* < 0.05) compared with WT.

The *OsAAE3* ortholog encode the peroxisomal co-enzyme synthetase in maize, sorghum and brachypodium. PODs mainly participate in a broad range of physiological processes, including lignin synthesis, reactive oxygen species (ROS) metabolism and programmed cell-death (PCD) (Kalsoom et al., 2015). ROS are essential to protect plants from various environmental stresses, while excessive accumulation of ROS causes damage to plant cells. Therefore, it remains unclear whether *OsAAE3* had effects on the POD activity during the ROS accumulation. We further extracted the total PODs from fresh leaves of *OsAAE3-OX* and wild-type plants when they grew under common conditions, and then examined the POD activity. The results suggested that the POD activity was ~50% lower in *OsAAE3-OX* plants compared with wild-type plants (Figure 7B). It has also been proved that intracellular POD activity is inhibited by the accumulated H_2_O_2_ level. Furthermore, RT-PCR data indicated that the expressions of POD synthesis-related genes at the mRNA level were significantly decreased in *OsAAE3-OX* plants (Figure 7A). In addition, we showed that the H_2_O_2_ level was significantly increased in *OsAAE3-OX* plants compared with wild-type plants (Figure 7C). These findings suggested that the over-expression of *OsAAE3* induced high concentration of H_2_O_2_ via repressing the POD activity, leading to hypersensitive response (HR) and PCD.

### Over-expression of *OsAAE3* suppresses the floret development

**Figure 8:**
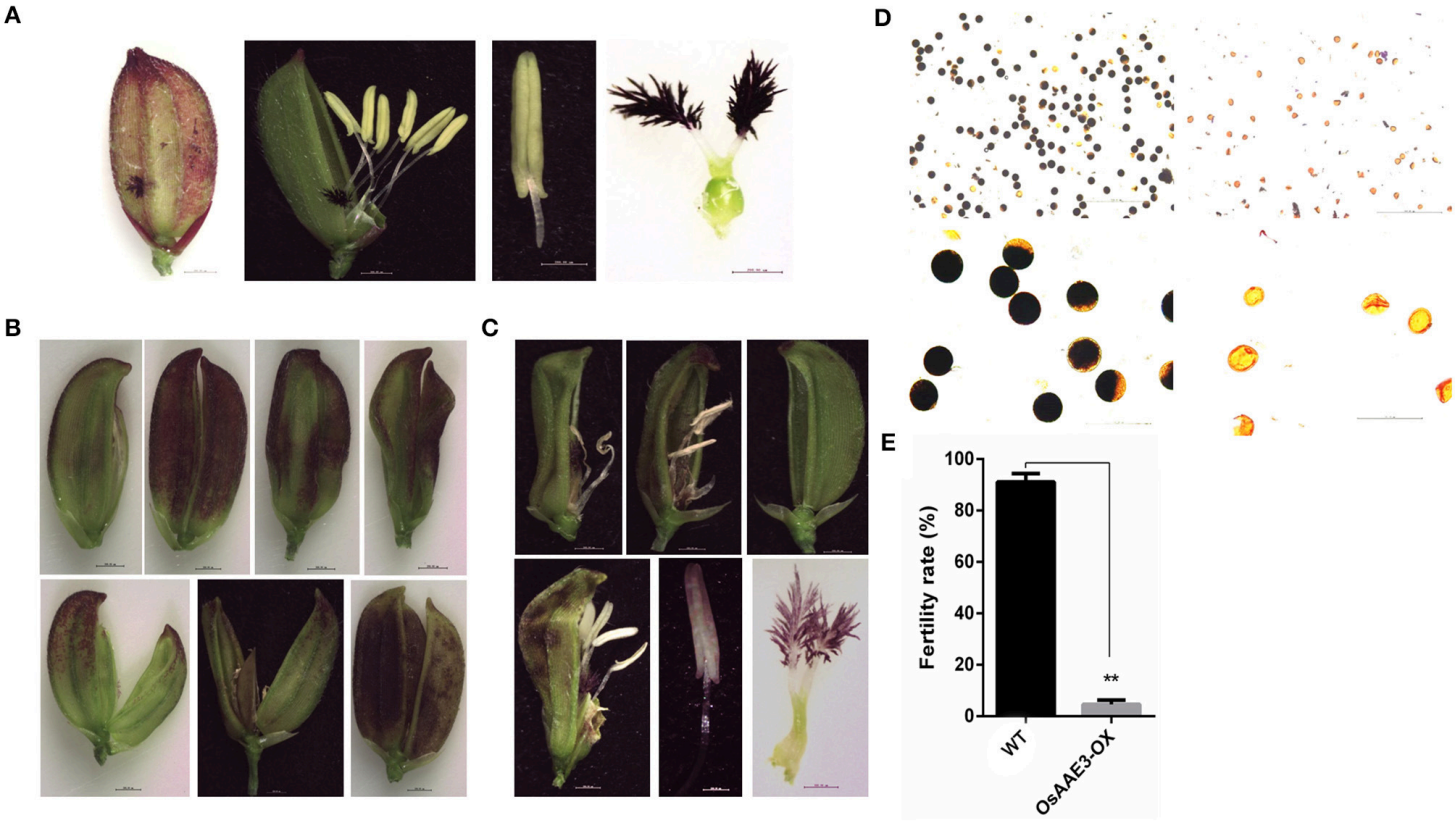
*****OsAAE3*** suppresses the floret development. (A)** Flower phenotype of wild-type plant at heading stage. **(B,C)** Microscopic analysis of *OsAAE3-OX* plants flower morphology at the heading stage. **(D)** Potassium iodide staining of pollen grains from the wild type and *OsAAE3* transgenic plants. **(E)** Fertility rate analysis of wild-type and *OsAAE3-OX*.

Floret is one of most important organ for rice development, which not only determines the growth time from the vegetative stage to the reproductive stage, but also offers sufficient seeds to extend the life of this species. In the present study, the over-expression of *OsAAE3* triggered several phenotypic alterations in the leaf, root, tiller number, flowering time, and flower development. The *OsAAE3-OX* plants obviously displayed an abnormal floret structure. The panicle heads and stalks hardly grew out from the leaf sheath at the heading stage, and each individual floret of the spikelet showed multiple abnormal and twisted glumes as well as increased number of empty glumes (Figures 8A,B). The interior palea was part of glume, and its development was intensively depressed and even disappeared. The spikelet also had an extra lodicule without expansion, leading to greatly impaired floret development.

Moreover, we examined the morphology of the floret structure in the *OsAAE3-OX* plants by microscopic analysis. The results revealed that the pistils and anthers also had abnormal morphology at the reproductive stage, and the stalk base of pistils was not expanded after flowering. The anther stalk suffered the external pressure from the twisted glume and showed the twisty phenotype (Figure 8C). Compared with wild-type plants, the anther of *OsAAE3-OX* plants was deficient and had a pale yellow color, showing wrinkled surface with larger pores (Figure 8C). Furthermore, we found that the basic viability of the pollen was affected by over-expression of *OsAAE3* (Figure 8D). The fertility rate of the *OsAAE3-OX* plants was significantly decreased to nearly 5% compared with 90% in wild-type plants (Figure 8E). These results suggested that *OsAAE3* over-expression exerted a great influence on the fertility of pollen *via* suppressing the anther development.

### *OsAAE3* represses the accumulation of total lignin

**Figure 9:**
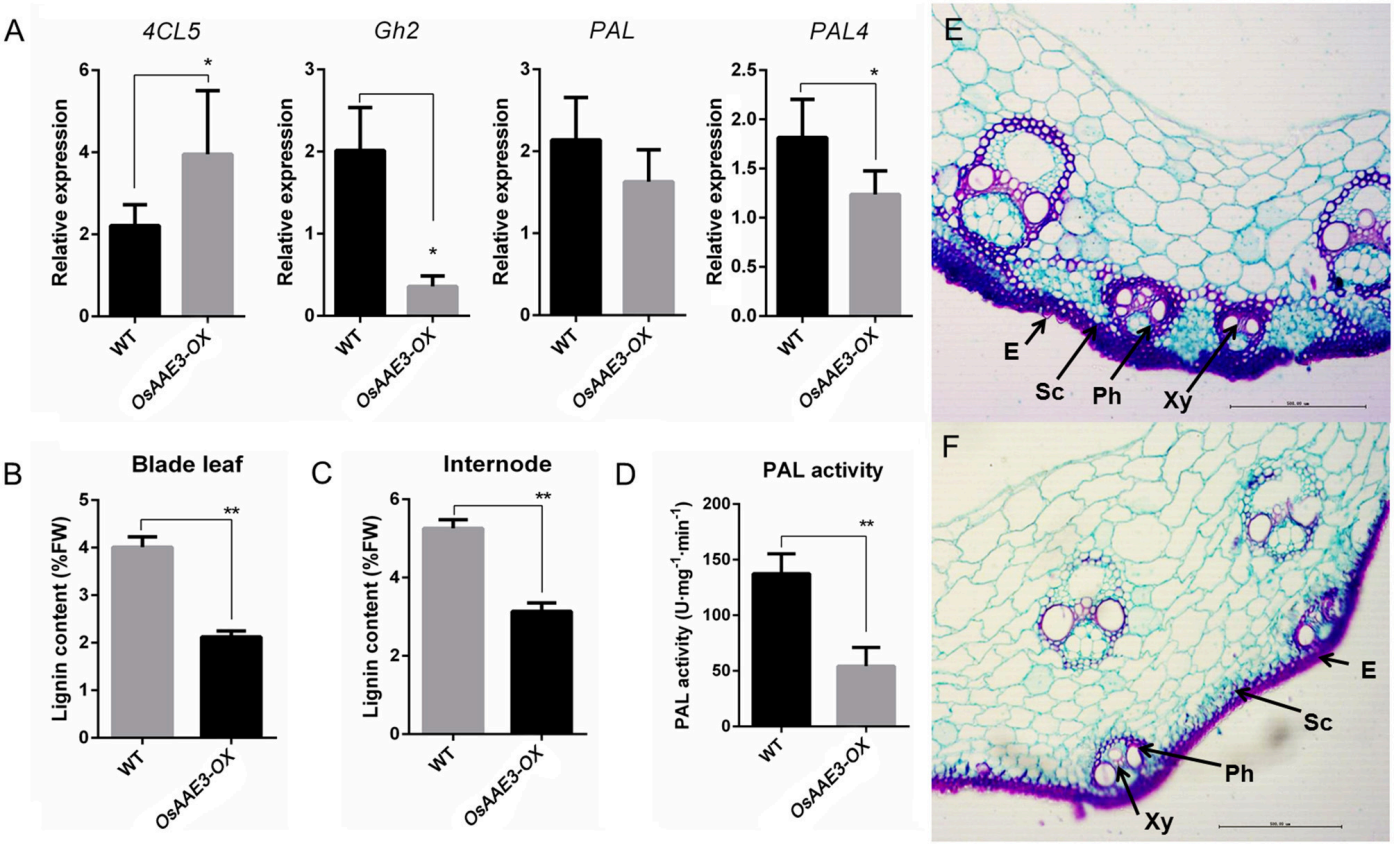
*****OsAAE3*** represses the accumulation of total lignin. (A)** Lignin biosynthesis-related genes expression in wild type and *OsAAE3-OX*. Values shown are means ± SD from three independent experiments, and asterisks indicate a significant difference according to the *t*-test (*P* < 0.05) compared with WT. **(B,C)** Total lignin content assay of blade leaf and internode in wild-type and *OsAAE3-OX*. Values shown are means ± SD (*n* = 10), and asterisks indicate a significant difference according to the *t*-test (*P* < 0.05) compared with WT. **(D)** PAL activity assay in wild-type and *OsAAE3-OX*. Values shown are means ± SD (*n* = 5), and asterisks indicate a significant difference according to the *t*-test (*P* < 0.05) compared with WT. **(E,F)** Lignin staining in wild-type and *OsAAE3-OX*. E, Epidermis; Ph, phloem; Sc, sclerenchyma cells; Xy, xylem.

*OsAAE3*-encoding protein contains a 4CL like domain, and the 4CL proteins function as key enzymes of phenylpropanoid metabolism in higher plants (Voelker et al., 2010). The question remains unclear whether *OsAAE3* regulated the lignin biosynthesis in rice. We first measured expressions of a subset of critical lignin synthesis genes in rice, including *4CL5* (Gui et al., 2011), *PAL, PAL4*, and *Gh2*. The results indicated that expressions of *PAL, PAL4*, and *Gh2* at the mRNA level were significantly decreased in *OsAAE3-OX* plants, while the expression of *4CL5* was obviously increased compared with wild-type plants (Figure 9A). Moreover, examination of PAL activity in wild-type and *OsAAE3-OX* showed that PAL activity was obviously decreased in OsAAE3 overexpression line (Figure 9D), directly suggesting that OsAAE3 repressed the PAL activity. Therefore, we concluded that *OsAAE3* over-expression promoted the expressions of phenylpropanoid catbolic genes and repressed the expressions of lignin biosynthesis genes.

Furthermore, we examined the content of total lignin in rice fresh blade leaf and internode at 2 months of age. The data demonstrated that *OsAAE3* over-expression affected the lignin biosynthesis (Figures 9B,C). In addition, the rice culms were sectioned and stained for lignin with safranin O/fast green regents. According to the structure of stained cell wall, we found that the lignin content was lower in *OsAAE3-OX* plants (Figures 9E,F). Taken together, up-regulation of *OsAAE3* obviously provoked the accumulation of 4CLs in cells, which predominantly disrupted the lignin biosynthesis.

## Discussion

### *OsAAE3* is closely related to *4CL*

*OsAAE3* belonged to the large superfamily of AMPBPs in rice, while the amino acid sequence of *AAE3* was not closely related to any other members. The most closely related proteins are 4CL like proteins in Arabidopsis, which exhibited 90% sequence similarity to *AAE3*, suggesting that *OsAAE3* had probably evolved into a subsidiary enzyme of 4CL (Figure S1). On the contrary, this clearly stated that *OsAAE3* was different from *Os4CLs* in biological function according to observed phenotype of *OsAAE3-OX* plants. 4CL ligates coenzyme A (CoA) with hydroxycinnamic acids, such as 4-coumaric and caffeic acids, into hydroxycinnamoyl-CoA thioesters, and these reactions accelerate the process of monolignol biosynthesis for lignification in plant cell walls (Chen et al., 2013). Although the major functions of *4CLs* have been broadly explored in Arabidopsis and rice, the detailed roles of 4CL-like proteins remain unclear (Taylor-Teeples et al., 2015). We believed that *OsAAE3* was most likely a 4CL, which mediated the phenylpropanoid metabolite. The phenylpropanoid pathway plays a critical role in the plant innate immunity system, which is responsible for offense-related compound biosynthesis, such as the flavonoids, total phenolics, and defense lignin (Zhang and Liu, 2015; Le Roy et al., 2016). Therefore, repression of the phenylpropanoid pathway through *OsAAE3* activation resulted in improved plant disease susceptibility. Both the expressions of *PAL1* and *PAL4* at the mRNA level were decreased in *OsAAE3-OX* plants, clearly supporting this hypothesis that *OsAAE3* was involved in the regulation of phenylpropanoid metabolism.

### *OsAAE3* negatively regulates the rice blast resistance

Fungus-induced rice blast is a potential threat to the rice production, which is characterized by sporadic and unpredictable outbreaks, leading to significant yield loss (Takatsuji, 2014). However, to protect themselves from pathogen invasion, plants have to effectively integrate extracellular and intracellular signals to activate the physiological and biochemical responses by enhancing the hormone defense pathway, switching off the plant growth and regulating the expressions of immunity-related genes and proteins (Lozano-Durán and Zipfel, 2015).

Interestingly, *M. oryzae* down-regulated the *OsAAE3* expression according to the database from transcriptome-proteome analysis, and we further confirmed such result by RT-PCR (Figure 1). However, *OsAAE3* over-expression improved the disease susceptibility (Figure 5A), therefore, we supposed that *OsAAE3* was a negative regulator in the complex immunity network of rice. Under the normal growth condition, plants are able to synthesize sufficient lignin for whole plant growth. In this case, *OsAAE3* utilized its 4CL-like structure to expand the chemical element phenylpropanoid and promote the lignin biosynthesis, and the balance between the phenylpropanoid metabolism and lignin biosynthesis was mediated by *OsAAE3* (Figures 9A,D). Therefore, rice had to switch off the *OsAAE3* expression under the *M. oryzae* invasion, and reduced *OsAAE3* activities were conducive to transformation of phenylpropanoid into defense-related compounds, such as plant hormone salicylic acid, phytoalexins and total phenolics (Liu et al., 2015). However, *OsAAE3* over-expression drastically disturbed the balance, and a large number of disease spots were gradually spread along with plant growth. In addition, *OsAAE3* over-expression also down-regulated the expressions of two *PR* genes, including *PR1a* and *PR10* (Figure 5B), which are tightly correlated with the onset of defense responses against a variety of fungal, viral, and bacterial pathogens (Liu et al., 2015).

Generally speaking, the disease spots on the leaf surface are correlated with the HR, and ROS accumulation may trigger the HR (Pottosin et al., 2014). Actually, plants scavenge destructive free radicals relying on robust antioxidant organelle class III peroxidases (*Prxs*) (Francoz et al., 2015). *Prxs* also serve as an important class of enzymes responsible for the stress-induced formation and degradation of ROS (Marjamaa et al., 2009). However, our survey indicated that *OsAAE3* over-expression depressed the POD activities and reduced the expressions of POD synthesis-related genes, thereby lowering POD activities and increasing the content of H_2_O_2_, which might be the major cause of cell death after *M. oryzae* inoculation (Figure 7).

Table S2 shows that we identified three potential proteins that also presented in the results of transcription-proteomics analysis, including the AMP-binding protein (LOC_Os04g58710), RRM recognition motif protein (LOC_Os03g25960), and protein of unknown function DUF1296 domain containing protein (LOC_Os01g47430). In addition, we also found that the Pik-H4 was most likely Pb1 to interact with WRKY45 (Inoue et al., 2013), and Pik-H4 was also associated with OsBIHD1 to balance the relation between the growth and blast resistance. These results suggested that the components Pik-H4-OsAAE3 were activated after recognizing the Avr-Pik. As a consequence, the Pik-H4 dissociated from the receptor complex, entered the nucleus and interacted with multiple transcription factors, and those transcription factors precisely controlled the switch of downstream target genes expression. Therefore, we believed that the *OsAAE3* abundance was regulated by the transcription factor under the pathogen invasion.

### *OsAAE3* involves in lignin synthesis pathway

A large part of host protection against invasion by fungal pathogens relies on a defensive system that is highly coordinated to prevent the spread of pathogens (Ye and Ma, 2016). Furthermore, enhanced deposition of lignin can provide a structural barrier against pathogen spread, and the toxic phenolic precursors produced during lignin biosynthesis or polymerization can directly inhibit pathogen multiplication and movement (Miedes et al., 2014). As a homolog of *OsAAE3* in Arabidopsis, *AAE3* encodes the oxalyl-CoA synthetase that is required for multiple physiology reactions, including oxalate degradation, seed development and defense against an oxalate-producing fungal pathogen. Here, our findings suggested that *OsAAE3* exerted a negative influence on lignin biosynthesis (Figure 9), and the content of lignin was significantly reduced in *OsAAE3-OX* plants. Meanwhile, GUS activity assay displayed that *OsAAE3* was expressed in the anther cell, and we speculated that *OsAAE3* over-expression caused the high concentration of H_2_O_2_. The accumulation of ROS might activate the process of PCD, and the fertility rate was obviously decreased in *OsAAE3-OX* plants compared with wild-type plants (Yi et al., 2016). Based on our histological observation of anther's section, we speculated that less accumulation of lignin repressed the tapetum development when mature pollen grain appeared, and this could be another reason of decreased fertility rate in *OsAAE3-OX* plants.

The questions remain unclear whether *OsAAE3* was involved in the oxalate degradation like its homolog in Arabidopsis, and whether *OsAAE3* induced dual-directional regulation between immunity network and plant growth pathways. If the answers were positive, there should be other transcriptional factors, which might regulate the *OsAAE3* expression after the immune activation by various pathogens. Our future work will focus on *OsAAE3*-modulated oxalate degradation, and we will identify the transcriptional regulators of *OsAAE3*. Taken together, our current study further expanded our knowledge in functions of AMBPs in plant development and the regulation of rice blast resistance.

## Author contributions

ZC, JW, TG, and HW conceived and designed the experiments. HL, ZG, WL, SK, SD, and FG performed the experiments. HL and DS analyzed the data and wrote the paper. MH, WX, GY, and YL revised the paper. All authors read and approved the final version of the paper.

### Conflict of interest statement

The authors declare that the research was conducted in the absence of any commercial or financial relationships that could be construed as a potential conflict of interest.
