# Supplementary material for: 4-Coumarate-CoA Ligase-Like Gene OsAAE3 Negatively Mediates the Rice Blast Resistance, Floret Development and Lignin Biosynthesis
Source: Front Plant Sci. 2017 Jan 10;7:2041. doi: 10.3389/fpls.2016.02041 (PMC5222848; doi:10.3389/fpls.2016.02041)
Supplement: Supplementary file 4 [file Image1.PDF]

4-Coumarate-CoA Ligase-Like Gene *OsAAE3* Negatively Mediates the Rice Blast Resistance, Floret Development， and Lignin Biosynthesis

Author names:

Hao Liu[1], Zhenhua Guo[2], Wei Liu[1], Shanwen Ke[3], Dayuan Sun[4], Shuangyu Dong[1], Fengwei Gu[1], Ming Huang[1], Wuming Xiao[1], Guili Yang[1], Yongzhu Liu[1], Tao Guo[1], Hui Wang[1], Jiafeng Wang[1]*, Zhiqiang Chen[1]*

Affiliations:

[1]National Engineering Research Center of Plant Space Breeding, South China Agricultural University, Guangzhou, 510642, Guangdong, China.

[2]Jiamusi Rice Research Institute of Heilongjiang Academy of Agricultural Sciences, Jiamusi, 154026, Heilongjiang, China.

[3]College of Agricultural, South China Agricultural University, Guangzhou, 510642, Guangdong, China.

[4]Plant Protection Research Institute Guangdong Academy of Agricultural Sciences/Guangdong Provincial key Laboratory of High Technology for Plant Protection, Guangzhou, 510640, China.

***Co-corresponding author**:

Jiafeng Wang,

E-mail: bcjfwang@gmail.com

Address: National Engineering Research Center of Plant Space Breeding, South China Agricultural University, Guangzhou, 510642, Guangdong, China.

*** Corresponding author**

Zhiqiang Chen,

E-mail: chenlin@scau.edu.cn

Address: National Engineering Research Center of Plant Space Breeding, South China Agricultural University, Guangzhou, 510642, Guangdong, China.

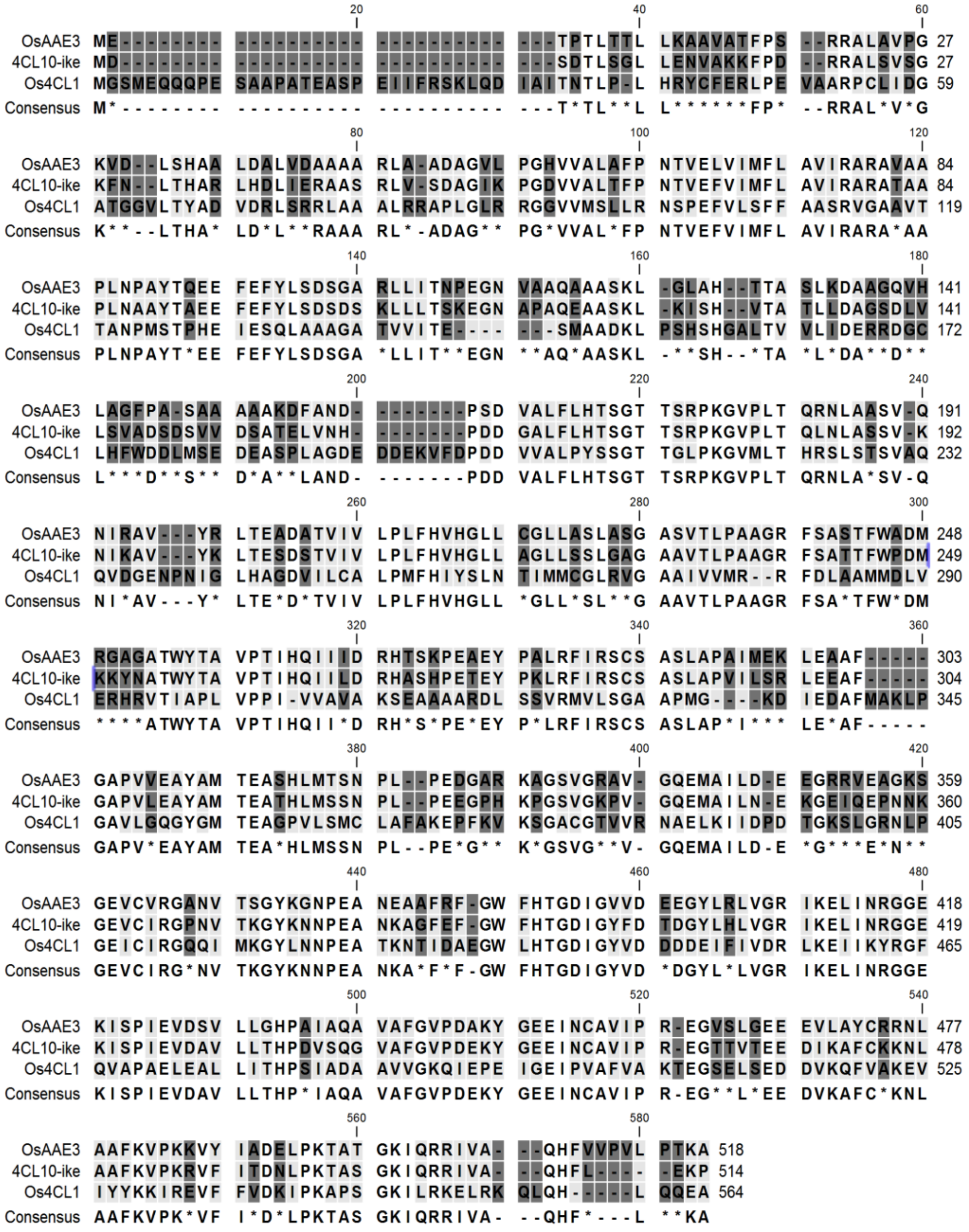

FIGURE S1. Amino acid sequence alignment of OsAAE3 and other 4CL from rice and Arabidopsis. Their gene accession numbers have been showed in figre 1.

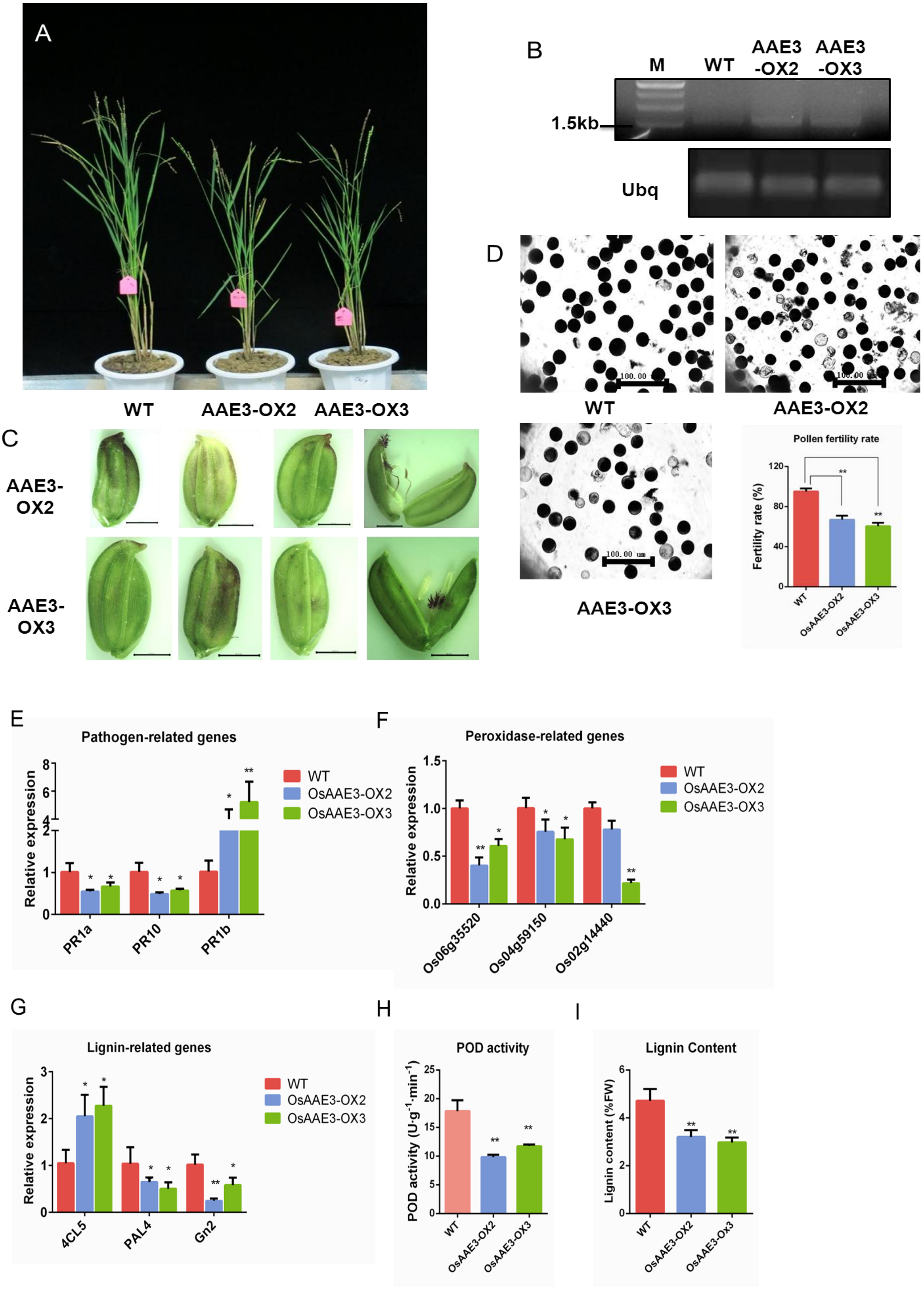

FIGURE S2. Phenotype of OsAAE3-OX2 and OsAAE3-OX3 transgenic plants. (A)

The Morphology of wild-type, OsAAE3-OX2, and OsAAE3-OX3 transgenic plants (T1 generation). The photograph was taken about 75 days after plant heading. (B) Expression analysis of OsAAE3 overexpression line measured by Semi-quantitative PCR, total RNA is 1ug, Ubq used as endogenous control. (C) Photographs of glume of OsAAE3-OX2 and OsAAE3-OX3, scale bar is 250μm. (D) Fertility rate analysis of OsAAE3-OX2 and OsAAE3-OX3. (E) The expression of pathogen-related genes in OsAAE3-OX2 and OsAAE3-OX3 plants. (F) The expression of peroxidase-related genes in OsAAE3-OX2 and OsAAE3-OX3 plants. (G) The expression of lignin-related genes in OsAAE3-OX2 and OsAAE3-OX3 plants. (H) POD activity assay in OsAAE3-OX2 and OsAAE3-OX3 plants. (I) Total lignin content assay in OsAAE3-OX2 and OsAAE3-OX3 plants. All the Values shown are means±SD (n=3), and asterisks indicate a significant difference according to the t-test ($P<0.05$) compared with WT.

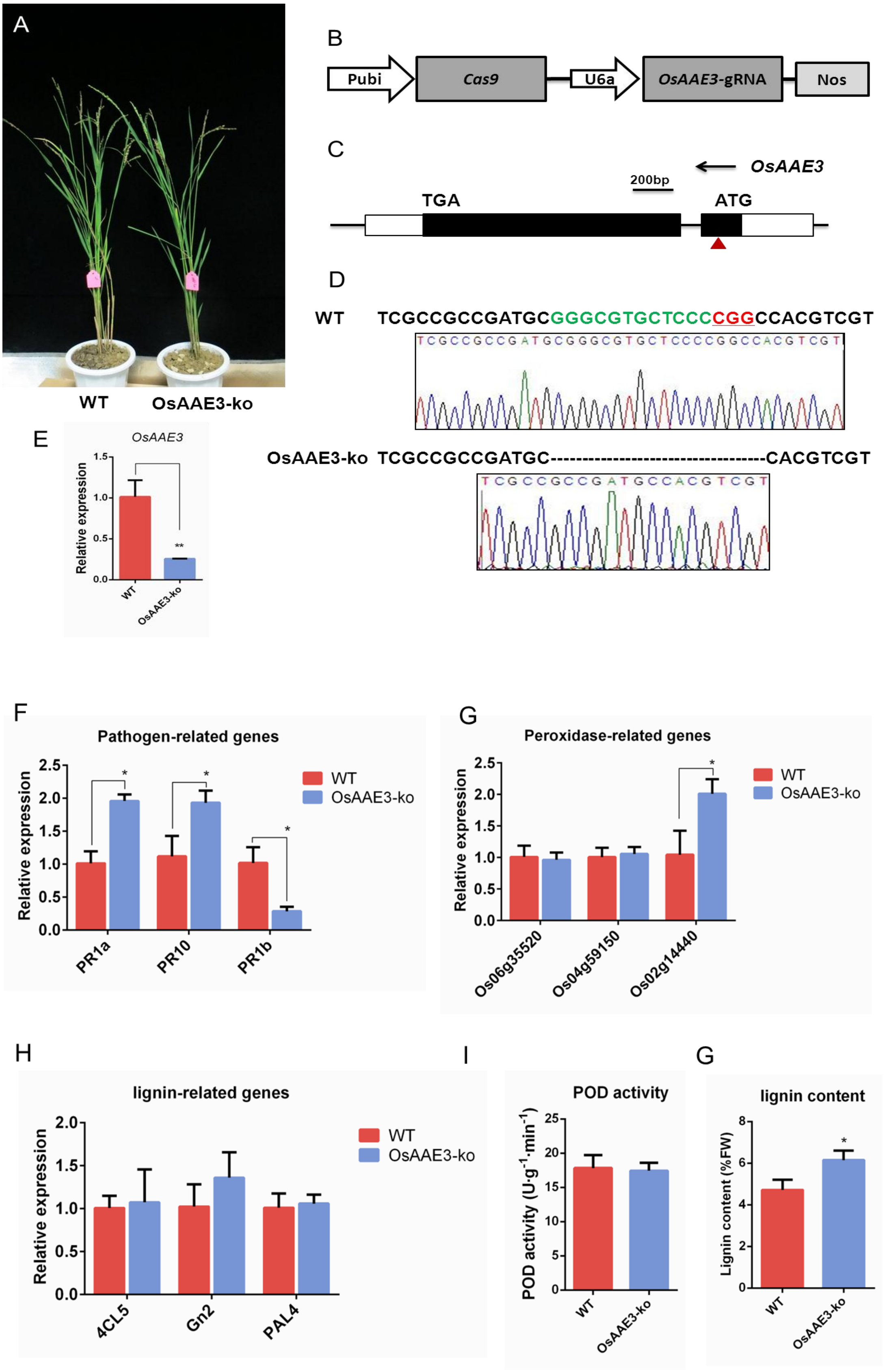
A
WT
OsAAE3-ko
B
Pubi
Cas9
U6a
OsAAE3-gRNA
Nos
C
OsAAE3
200bp
TGA
ATG
D
WT TCGCCGCCGATGCGGGCGTGCTCCCCGGCCACGTCGT
OsAAE3-ko TCGCCGCCGATGC----------------------------------CACGTCGT
E
OsAAE3
Relative expression
1.5
1.0
0.5
0.0
**
WT
OsAAE3-ko
F
Pathogen-related genes
Relative expression
2.5
2.0
1.5
1.0
0.5
0.0
*
WT
OsAAE3-ko
PR1a
PR10
PR1b
G
Peroxidase-related genes
Relative expression
Os06g35520
Os04g59150
Os02g14440
H
lignin-related genes
Relative expression
4CL5
Gn2
PAL4
I
POD activity
POD activity (U·g-1·min-1)
25
20
15
10
5
0
G
lignin content
Lignin content (%FW)
8
6
4
2
0

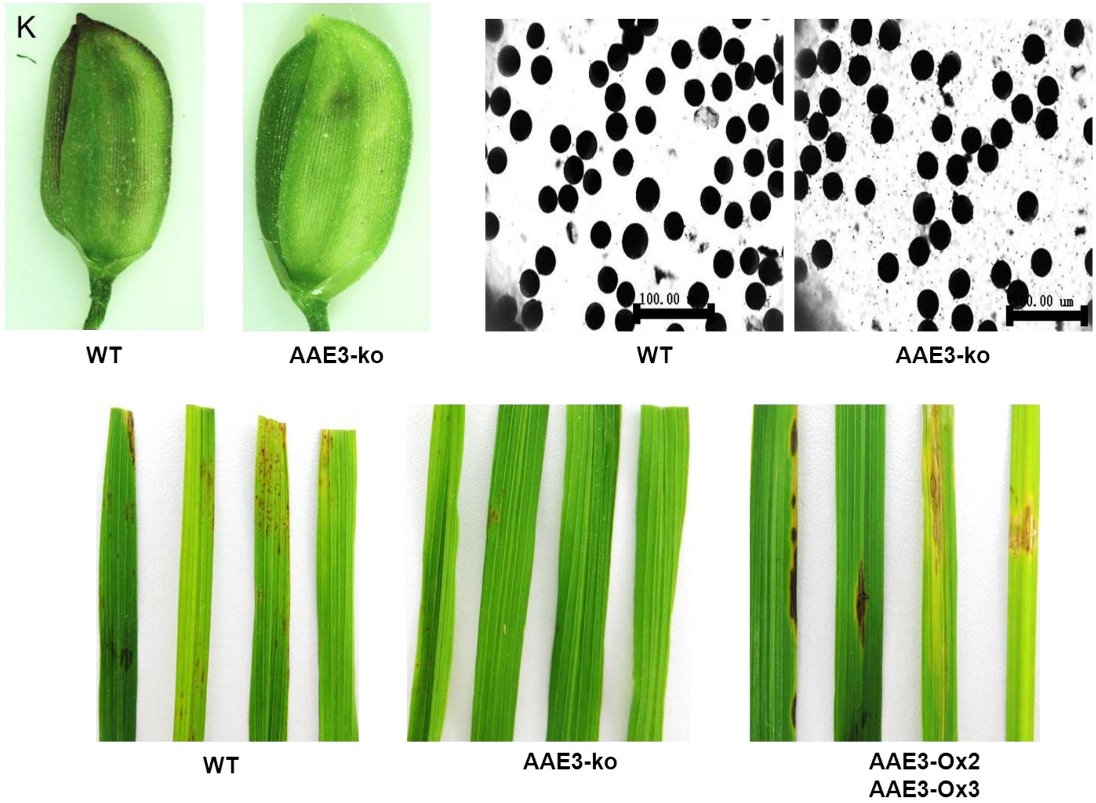

FIGURE S3. Phenotype of OsAAE3-ko transgenic plant. (A) The Morphology of wild-type, OsAAE3-ko transgenic plants (T0 generation). The photograph was taken about 75 days after plant heading. (B) Schematic structure of OsAAE3-ko transgenic vectors construction. (C) Target sequence alignment of OsAAE3 CRISPR/Cas9 splice sites. The red sequence of wild-type is the PAM sequence, and the green sequence is a paired gRNA binding site of wild-type. (D) Expression analysis of OsAAE3 knock-out line measured by real-time PCR compared with wild-type. (F-H) The relative expression of the pathogen-related genes, peroxidase-related genes, and lignin-related genes in OsAAE3-ko transgenic plant, respectively. (I) POD activity assay in OsAAE3-ko. (G) Total lignin content assay in OsAAE3-ko plants. All the Values shown are means±SD (n=3), and asterisks indicate a significant difference according to the t-test ($P<0.05$) compared with WT. (K) The Morphology of glume, pollen, and blast resistance in OsAAE3-ko plant.
